# Lower limb ischemia caused by resuscitative balloon occlusion of aorta

**DOI:** 10.1186/s40792-016-0260-4

**Published:** 2016-11-10

**Authors:** Yohei Okada, Hiromichi Narumiya, Wataru Ishi, Iiduka Ryoji

**Affiliations:** Department of Emergency and Critical Care Medicine, Japanese Red Cross Society Kyoto Daini Hospital, 355-5 Haruobicho Kamigyoku, Kyoto, 602-8026 Japan

**Keywords:** Balloon occlusion, Hemorrhagic shock, Ischemia, Near-infrared spectroscopy, Blunt abdominal injury, IABO, REBOA, Sheath, Femoral artery

## Abstract

**Background:**

Resuscitative endovascular balloon occlusion of the aorta (REBOA) is an emergency procedure to manage severe hemorrhagic shock from torso injury but can cause severe ischemia of the lower extremities. However, lower extremity ischemia occurring as a complication of REBOA has been rarely reported. We describe the severe lower extremity ischemia caused by REBOA with a 12-Fr sheath in a small-built patient.

**Case representation:**

The patient was a 16-year-old male who developed severe hemorrhagic shock due to abdominal blunt trauma. Following REBOA with a 12-Fr sheath on the right femoral artery, an emergency laparotomy and angiography to control the hemorrhage were performed. Twenty-eight hours after admission, suspecting lower extremity ischemia and compartment syndrome, we removed the sheath with a manual maneuver and performed fasciotomy. The limb ischemia was thus partially resolved. However, amputation was necessary because of ischemic necrosis on day 32. Our patient was physically small, and the diameter of his femoral artery on the contralateral site of sheath placement was also small. Therefore, disproportion of the sheath and femoral artery sizes may have caused the ischemic complication.

**Conclusion:**

Our experience highlights the importance of appropriate size selection for the sheath in line with the target vessel. We also recommend postoperative monitoring of limb perfusion in such cases with the use of near-infrared spectroscopy to facilitate the early detection of ischemia.

## Background

Resuscitative endovascular balloon occlusion of the aorta (REBOA) is an emergency procedure for the management of severe hemorrhagic shock caused by torso injury. This intervention can cause severe ischemia of the lower extremities [1]. However, ischemic complication of the lower extremities after REBOA has been rarely reported. We describe the severe lower extremity ischemia directly attributable to REBOA.

## Case presentation

**Fig. 1:**
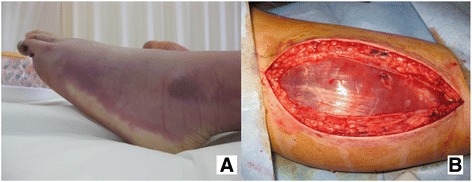
**a** Swelling and paleness of the left lower extremity. **b** Photograph showing fasciotomy for alleviation of acute compartment syndrome

A previously healthy 16-year-old male with blunt abdominal injury sustained in a road traffic accident was referred to our department. On arrival, he was in severe hemorrhagic shock, and his vital signs were as follows: heart rate 160 bpm, blood pressure unmeasurable, respiratory rate 27/min, and Glasgow Coma Scale E3V4M6. His chest X-ray revealed no apparent hemothorax and pneumothorax, and pelvic X-ray revealed no unstable fracture of the pelvic ring. Focused assessment with sonography for trauma was positive; thus, we suspected intra-abdominal hemorrhage. The arterial blood gas assessment showed metabolic acidosis, and the laboratory test on admission revealed coagulopathy (Table 1). The fluid resuscitation (crystalloid 1000 ml bolus infusion) improved his blood pressure to 71/37 mmHg. REBOA was placed with a 12-Fr sheath on the right femoral artery by the Seldinger method without sonography 10 min after admission, and the balloon placement at zone 1 was confirmed by chest X-ray. The patient was prepared for an emergency laparotomy 25 min after admission. During the surgery, we found splenic rupture, stomach perforation, hepatic injury, rectal and omental injury, and splenectomy; temporary repair of the injury, gauze packing, and vacuum packing closure were performed as damage control measures (operation time 65 min, intraoperative blood loss 2470 g). REBOA was performed for 25 min with total occlusion and deflated after the control of major bleeding. Postoperative enhanced computed tomography (CT) revealed extravasation from the left kidney and posterior segment of the liver. Thus, we performed angiography for embolization. After admission to the intensive care unit, coagulopathy and hypothermia were corrected with massive blood transfusion; however, his vital signs continued to remain unstable. A chest drainage tube was inserted for left hemothorax removal. Due to increased amount of blood discharge from the chest, we performed exploratory thoracotomy and re-laparotomy. During the second surgery, the blood leakage was found to originate from the hepatic injury and passed through the diaphragmatic injury into the thoracic cavity; we then arrested the bleeding. Fourteen hours after admission, the patient became stable. However, we retained the sheath for a potential repeat of REBOA in the event of re-bleeding. Twenty-eight hours after admission, swelling and paleness were observed in his right lower leg (Fig. 1a). Suspecting lower extremity ischemia and compartment syndrome, we removed the sheath with a manual maneuver and performed fasciotomy for the lower leg (Fig. 1b). The color of the right lower limb was thus partially resolved, and we guessed the ischemic change was improved. However, amputation was necessary because of ischemic necrosis on day 32. On day 81, he was transferred to a community hospital for rehabilitation.

**Table 1:** Laboratory test and atrial blood gas assessment on admission

CBC	
WBC	9200/μl
RBC	297 × 104/μl
Hb	8.8 g/dl
Ht	25.2%
Plt	14.9 × 104/μl
Chemistry	
AST	83 U/l
ALT	62 U/l
CPK	511 U/l
Cr	0.89 mg/dl
BUN	15.7 mg/dl
Na	138 meq/l
K	3.3 meq/l
Cl	106 meq/l
CRP	0.01 mg/dl
Coagulation	
PT–INR	1.56
APTT	47.8 s
Fib	140 mg/dl
Atrial blood gas^a^	
pH	7.244
PaCO_2_	37.8 mmHg
PaO_2_	381 mmHg
HCO_3_^−^	15.8 mmol/l
Base excess	−10.3 mmol/l
Lac	6.8 mmol/l

^a^O_2_ 10-l non-rebreather mask

### Discussion

We describe the severe ischemic complication of the lower extremities associated with sheath placement for REBOA. Potential ischemic complications of REBOA include acute kidney injury, vascular injury, bowel ischemia, spinal cord ischemia, and lower extremity ischemia [1]. However, incidence of these complications is very rare [2].

**Fig. 2:**
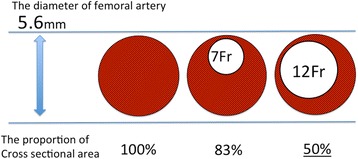
Illustration of an artery model with in situ sheath. The size of the femoral artery in our patient was 5.6 mm. This figure illustrates a 5.6-mm artery model with sheath sizes of 7-Fr and 12-Fr. The proportion of cross-sectional vascular area with the use of 12-Fr sheath reduces by 50 % of the original cross-sectional area

**Fig. 3:**
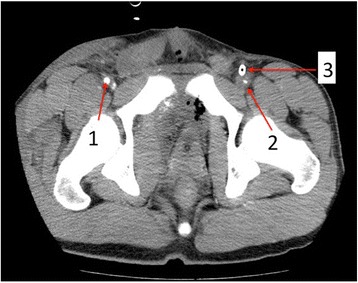
Enhanced computed tomography imaging after the primary surgery. *Arrow 1*: right femoral artery (size 5.6 mm). *Arrow 2*: left femoral artery. It was not enhanced due to sheath placement. *Arrow 3*: 12-Fr sheath placed in the left femoral artery. Lack of enhancement of the left femoral artery indicates lack of blood flow

**Fig. 4:**
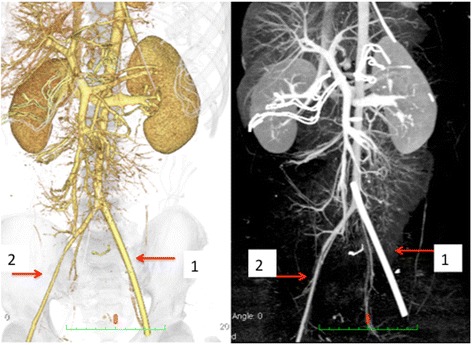
Three-dimensional computed angiogram performed after the primary surgery. *Arrow 1*: 12-Fr sheath in the left femoral artery. *Arrow 2*: right external iliac-femoral artery. Contralateral (right) external iliac-femoral artery appears thinner than the sheath placed in the left

We believe that the use of large-sized sheaths for REBOA is a critical risk factor for lower extremity ischemia. In a prospective REBOA case registry, 74 % (34/46) of all REBOA interventions were performed using a 12- or 14-Fr sheath [2]. Although 7-Fr sheaths for REBOA are currently available and frequently used in Japan, this was not the case at the time of treatment of this patient. Therefore, we had to use a 12-Fr sheath. As blood flow is inversely proportional to the vessel cross-sectional area [3], it is acceptable that large-sized sheaths may decrease blood flow to the extremities. For example, the cross-sectional area of the blood flow was shown to reduce by 50 % in a model wherein a 12-Fr sheath (diameter 4 mm) is inserted in an artery with 5.6-mm diameter (the size of the femoral artery in our case) (Fig. 2). In a patient who underwent an attempted transfemoral transcatheter aortic valve replacement, a high sheath femoral artery diameter ratio and sheath femoral artery area ratio estimated on CT angiogram were reported as significant predictors of vascular complications [4]. Our patient was physically small (156 cm), and the diameter of his femoral artery on the contralateral site of sheath placement was also small (5.6 mm), according to the CT imaging after the primary surgery (Figs. 3 and 4). Therefore, disproportion of the sheath and femoral artery sizes may have caused the ischemic complication. We propose that the use of large-sized sheaths in small arteries increases the risk for lower extremity ischemia. Additionally, hypotension and occlusion of the aorta with REBOA may further aggravate the risk of ischemic complications due to decrease in lower extremity arterial flow. A retrospective study showed that 7-Fr sheaths for REBOA can be safely used [1]; however, even 7-Fr sheaths should be used with caution especially in small-built patients such as women or adolescents.

We believe that decreasing the duration of sheath placement and aorta occlusion may also help avert lower extremity ischemia. The reported median duration of sheath placement in a study was 28 (18–45) h, which is equal to the duration in our case (28 h) [5]. In an animal experiment, 60 min of REBOA was well tolerated and recoverable [6], and the median time of total balloon occlusion was reported as 26 (10–35) min, which is similar to that in our case [5]. Although the duration of sheath replacement and aorta occlusion in our case were believed to be tolerable, had we decreased the duration of sheath placement and aortal occlusion to as short as possible, the complication may have been averted.

Monitoring of lower extremity perfusion is essential to detect ischemia. We relied on visual examination and Doppler study for this purpose; however, we could not avoid the ischemic complication as both these methods lack objectivity and are prone to inter-observer variability. This probably contributed to the delay in the detection of ischemia. Near-infrared spectroscopy (NIRS) is reported as a good monitoring tool to detect lower extremity ischemia and compartment syndrome [7, 8]. Use of NIRS for this purpose should be considered.

## Conclusions

We described severe lower extremity ischemia occurring as a complication of REBOA using 12-Fr sheath in a small-built patient. We highlighted the importance of appropriate size selection for the sheath in line with the target vessel. We also recommend postoperative monitoring of limb perfusion in such cases with the use of NIRS to facilitate early detection of ischemia.

## Abbreviations

CT: Computed tomography
NIRS: Near-infrared spectroscopy
REBOA: Resuscitative endovascular balloon occlusion of the aorta
